# Sialylation of EGFR by the ST6Gal-I sialyltransferase promotes EGFR activation and resistance to gefitinib-mediated cell death

**DOI:** 10.1186/s13048-018-0385-0

**Published:** 2018-02-05

**Authors:** Colleen M. Britain, Andrew T. Holdbrooks, Joshua C. Anderson, Christopher D. Willey, Susan L. Bellis

**Affiliations:** 1 Department of Cell, Developmental, and Integrative Biology, University of Alabama at Birmingham, 350 McCallum Building, 1918 University Blvd, Birmingham, AL 35294 USA; 2 Department of Radiation Oncology, University of Alabama at Birmingham, 1700 6th Avenue South, Birmingham, AL 35233 USA

**Keywords:** β-galactoside α2-6 sialyltransferase 1 (ST6GAL1), Glycosylation, Epidermal growth factor receptor (EGFR) cell signaling, Gefitinib, Tumor cell biology, Kinomics, Tyrosine kinase

## Abstract

**Background:**

The ST6Gal-I sialyltransferase is upregulated in numerous cancers, and high expression of this enzyme correlates with poor patient prognosis in various malignancies, including ovarian cancer. Through its sialylation of a select cohort of cell surface receptors, ST6Gal-I modulates cell signaling to promote tumor cell survival. The goal of the present study was to investigate the influence of ST6Gal-I on another important receptor that controls cancer cell behavior, EGFR. Additionally, the effect of ST6Gal-I on cancer cells treated with the common EGFR inhibitor, gefitinib, was evaluated.

**Results:**

Using the OV4 ovarian cancer cell line, which lacks endogenous ST6Gal-I expression, a kinomics assay revealed that cells with forced overexpression of ST6Gal-I exhibited increased global tyrosine kinase activity, a finding confirmed by immunoblotting whole cell lysates with an anti-phosphotyrosine antibody. Interestingly, the kinomics assay suggested that one of the most highly activated tyrosine kinases in ST6Gal-I-overexpressing OV4 cells was EGFR. Based on these findings, additional analyses were performed to investigate the effect of ST6Gal-I on EGFR activation. To this end, we utilized, in addition to OV4 cells, the SKOV3 ovarian cancer cell line, engineered with both ST6Gal-I overexpression and knockdown, as well as the BxPC3 pancreatic cancer cell line with knockdown of ST6Gal-I. In all three cell lines, we determined that EGFR is a substrate of ST6Gal-I, and that the sialylation status of EGFR directly correlates with ST6Gal-I expression. Cells with differential ST6Gal-I expression were subsequently evaluated for EGFR tyrosine phosphorylation. Cells with high ST6Gal-I expression were found to have elevated levels of basal and EGF-induced EGFR activation. Conversely, knockdown of ST6Gal-I greatly attenuated EGFR activation, both basally and post EGF treatment. Finally, to illustrate the functional importance of ST6Gal-I in regulating EGFR-dependent survival, cells were treated with gefitinib, an EGFR inhibitor widely used for cancer therapy. These studies showed that ST6Gal-I promotes resistance to gefitinib-mediated apoptosis, as measured by caspase activity assays.

**Conclusion:**

Results herein indicate that ST6Gal-I promotes EGFR activation and protects against gefitinib-mediated cell death. Establishing the tumor-associated ST6Gal-I sialyltransferase as a regulator of EGFR provides novel insight into the role of glycosylation in growth factor signaling and chemoresistance.

## Background

It has long been known that tumor cells display an altered profile of cell surface glycans, however the functional role of glycosylation in regulating tumor cell behavior remains poorly-understood. The changes in tumor glycosylation are not random; instead, a select subset of glycans is consistently enriched in cancer cells. One of these elevated glycan structures is α2-6 linked sialic acid, which is added to *N*-glycosylated proteins by the ST6Gal-I sialyltransferase [1–3]. ST6Gal-I is upregulated in numerous cancers including ovarian, pancreatic, colon and breast [4–8], and high ST6Gal-I expression correlates with poor patient outcomes in several types of malignancies [5–8].

One of the central questions regarding ST6Gal-I’s pro-tumorigenic activity is how changes in surface sialylation influence intracellular signaling cascades to modulate tumor cell behavior. We and others have reported that ST6Gal-I regulates the structure and function of a specific cohort of membrane receptors. As examples, ST6Gal-I-mediated sialylation of the β1 integrin drives tumor cell migration and invasion [9–12], whereas α2-6 sialylation of both the Fas and TNFR1 death receptors prevents apoptosis by blocking ligand-induced receptor internalization [13, 14]. ST6Gal-I-dependent sialylation also plays a prominent role in regulating the oligomerization of multiple receptors including CD45 [15] and PECAM [16]. Through its collective actions on diverse receptors, ST6Gal-I functions as a master regulator to control cell phenotype. In cancer cells, the upregulation of ST6Gal-I promotes hallmark cancer stem cell (CSC) behaviors including tumorspheroid growth, self-renewal, tumor-initiating potential and resistance to chemotherapy [4, 5, 17–19].

In the present study we identify another important receptor regulated by ST6Gal-I, the receptor tyrosine kinase, EGFR. OV4 ovarian cancer cells with enforced ST6Gal-I expression were subjected to an unbiased kinomics assay, which revealed that EGFR was one of the most differentially activated kinases in cells with upregulated ST6Gal-I. Specifically, EGFR tyrosine kinase activity was markedly enhanced in cells with high ST6Gal-I expression. Based on the kinomics results, we developed several cell model systems with either ST6Gal-I overexpression or knockdown to establish that EGFR is directly α2-6 sialylated by ST6Gal-I. Significantly, we find that ST6Gal-I-mediated sialylation of EGFR stimulates both the basal and EGF-induced activation of EGFR. Furthermore, α2-6 sialylation of EGFR regulates the viability of cells exposed to the EGFR inhibitor, geftinib. These results not only establish a new tumor-promoting function for ST6Gal-I, but also more broadly illuminate the importance of tumor glycans in fundamental tumor cell survival pathways.

## Methods

### Cell culture

For routine maintenance of cell lines, cells were grown in DME/F12 (OV4) or RPMI (SKOV3 and BxPC3) media supplemented with 10% fetal bovine serum (FBS – Atlanta Biologicals) and 1% antibiotic/antimycotics (Invitrogen). Cells were transduced with lentivirus encoding either the human ST6Gal-I gene (Genecopoeia) or shRNA against ST6Gal-I (Sigma, TRCN00000035432, sequence CCGGCGTGTGCTACTACTACCAGAACTCGAGTTCTGGTAGTAGTAGCACACGTTTTTG). Polyclonal populations of stably-transduced cells were isolated by puromycin selection. Overexpression or knockdown of ST6Gal-I was verified via immunoblot using anti-ST6Gal-I goat polyclonal antibody (R&D Systems, AF5924).

### Kinomics assay

OV4 EV or OE cells were lysed by adding pre-chilled M-PER Mammalian Protein Extraction Reagent (Thermo-Scientific) containing protease and phosphatase inhibitors (Thermo Scientific). After a 30 min incubation on ice, the lysate was centrifuged at 14,000 rpm at 4 °C, and the supernatant immediately collected and stored at − 80 °C. Global kinase activity (kinomic) profiling was performed in the UAB Kinome Core (www.kinomecore.com) using the PamStation®12 platform (PamGene, BV, The Netherlands) as previously described [20–25]. Briefly, lysates were loaded onto wells of 2% BSA blocked PamChips specific for the kinome analyzed – 15 μg lysate for the protein tyrosine kinase (PTK) chip and 2 μg lysate for the serine threonine kinase (STK) chip. Lysates were loaded along with standard kinase buffer (PamGene) containing ATP and FITC-labeled antibodies for detection of phosphorylated substrate probes. Both kinetic and end of reaction (end-level) peptide substrate phosphorylation image capture data was collected with Evolve software (PamGene) and analyzed using the BioNavigator (v. 6.2, PamGene). Upstream kinase prediction was performed using the UpKin upstream kinase prediction tool (PamGene) that calculates a normalized kinase statistic score and specificity score using data from the public phosphonet database (www.phosphonet.ca) to identify highly altered kinases that are displayed in bar graph and volcano plots [21, 22].

### Immunoblotting

Cells were serum deprived for 2 h using media containing 1% FBS prior to treatment with 100 ng/mL rhEGF (R&D, 236-EG). Cells were treated for indicated times and lysed using radioimmune precipitation assay (RIPA) buffer supplemented with protease and phosphatase inhibitors. Total protein concentration was measured by BCA (Pierce). Samples were resolved by SDS-PAGE and transferred to polyvinylidene difluoride membranes. Membranes were incubated with 5% nonfat dry milk in TBS containing 0.1% Tween-20 (TBST). Immunoblots were probed with antibodies for p-EGFR (Y-1068, Cell Signaling Technology, cat #3777), or total EGFR (Cell Signaling Technology, cat #4267), followed by incubation with appropriate HRP-conjugated secondary antibodies (Cell Signaling Technologies). Protein loading was verified using anti-β-tubulin (Abcam, ab21058). Protein was detected by enhanced chemiluminescence using the ECL substrate from Pierce (cat# 32106). To visualize basal p-EGFR levels, which are lower than levels of EGF-induced p-EGFR, we optimized blotting conditions by increasing the total amount of protein loaded (> 40 μg) and prolonging film exposure times. We also used a more sensitive ECL reagent for the basal p-EGFR blots (SuperSignal West Dura from BioRad cat# 179-5060). In addition to immunoblotting for p-EGFR and total EGFR, OV4 cells were immunoblotted for total levels of tyrosine phosphorylation using an HRP-conjugated antibody against phospho-tyrosine (BD Biosciences, cat #610011). Immunoblotting for p-EGFR was performed using three independently-prepared cell lysates, and densitometric quantification of bands from at least three independent blots was achieved using ImageJ software. All bands were normalized to their respective β-tubulin loading controls. Student’s t test was employed to determine significance (*p* < 0.05).

### SNA precipitation assay

250 μg of cell lysate was incubated with 150 μg of SNA-agarose (Vector Labs, cat# AL-1303). Samples were incubated at 4 °C overnight on a rotator. α2-6 sialylated proteins were then precipitated by centrifugation and washed 3 times with ice cold PBS. Precipitates were resolved by SDS-PAGE and immunoblotted for EGFR as described above.

### Caspase 3/7 luminescence assay

Cells were seeded at equal densities into culture plates and allowed to adhere overnight. Prior to gefitinib treatment, cells were serum deprived in 1% FBS-containing media for 2 h and then treated with 1 μM gefitinib (Selleckchem, cat #S1025) for 24-72 h. Reconstituted Caspase-Glo 3/7 assay reagent (Promega, cat #G8093) was then added into each well, mixed via orbital shaker, and incubated at room temperature for 45 min. Luminescence was quantified with a BioTek Synergy H1 plate reader. The values represented were normalized to the caspase value for untreated cells. At least two independent experiments were conducted for each cell line.

## Results

### Kinomics assays reveal that ST6Gal-I overexpression switches signaling to favor tyrosine kinase activation

**Fig. 1:**
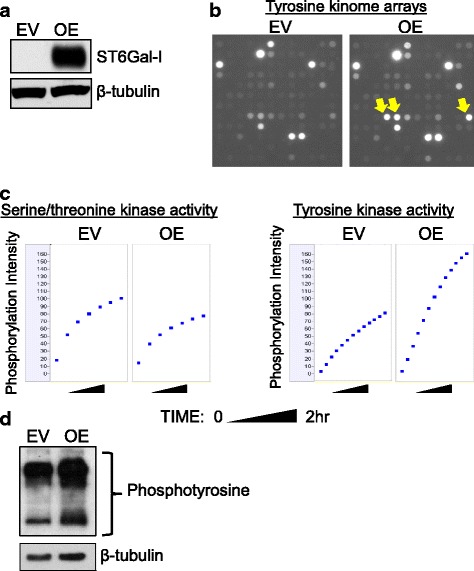
ST6Gal-I promotes an increase in overall tyrosine kinase activity. **a.** OV4 cells were stably transduced with lentivirus encoding ST6Gal-I, and ST6Gal-I overexpression (OE) was confirmed by immunoblotting (EV = empty vector control). **b.** Whole array image capture at final prewash cycle number 92 of PTK array illustrating changes in tyrosine phosphorylation with qualitatively selected altered spots (yellow arrows) increased in ST6Gal-I OV4 OE cells as compared to EV cells. **c.** Whole chip comparative OE and EV array-mean phosphorylation intensity (y axis per cell) over time in the kinetic/prewash cycles (x axis per cell) for both the STK (left 2 panels) and PTK (right two panels) arrays. **d.** EV or OE cells were immunoblotted with an anti-phosphotyrosine antibody

The OV4 ovarian cancer cell line is one of the few cancer lines that lacks detectable ST6Gal-I protein. To evaluate the role of ST6Gal-I in regulating kinase activity, ST6Gal-I was stably overexpressed (OE) in OV4 cells using a lentiviral vector (Fig 1a). An empty vector (EV) control cell line was also generated. As previously reported [26], OV4 OE cells have increased surface α2-6 sialylation relative to EV cells, as measured by SNA labelling and flow cytometry. SNA is a lectin that specifically recognizes α2-6 sialic acids.

To screen for potential changes in cell signaling consequent to forced ST6Gal-I expression, EV and OE cells were subjected to a kinomics assay. As shown in Fig. 1b, EV and OE cells displayed noticeable differences in the phosphorylation of select substrates (arrows), suggesting that ST6Gal-I modulates the activity of a distinct subset of kinases. Interestingly, OE cells exhibited a modest decrease in the net activity of serine/threonine kinases, compared with EV, cells (Fig. 1c, left panel), whereas OE cells had substantially increased tyrosine kinase activity (Fig. 1c, right panel). Enhanced tyrosine kinase activity in the OE line was confirmed by immunoblotting whole cell lysates with an anti-phosphotyrosine antibody that detects global changes in tyrosine kinase activity (Fig 1d).

**Fig. 2:**
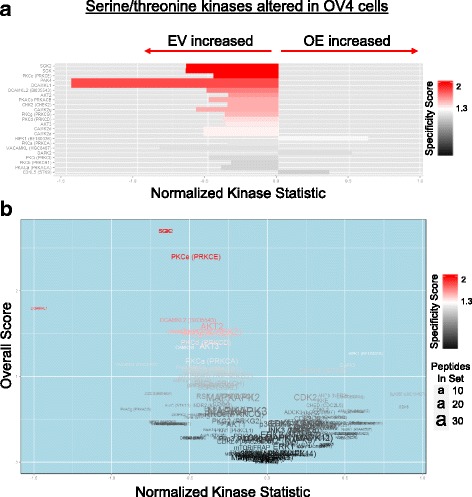
ST6Gal-I overexpression attenuates serine/threonine kinase activity. Bar and volcano plots of kinases with altered STK activity in OV4 OE vs. EV cells. **a.** Bar plot of serine/threonine kinases identified with the BioNavigator UpKin STK PamApp v14.0 (PamGene) scored as increased (rightward) or decreased (leftward) in OE relative to EV. Length of bar indicates extent of change (KSTAT; Kinase Statistic) and color of bar indicates specificity of each kinase to the predicted phosphosites used to measure its respective activity. **b.** In the volcano plot, kinases are similarly scored as increased or decreased (y axis) in OE relative to EV, colored by specificity. A combined KSTAT + specificity score (x axis) is denoted, with text size indicating the number of seed peptides used to identify that kinase

**Fig. 3:**
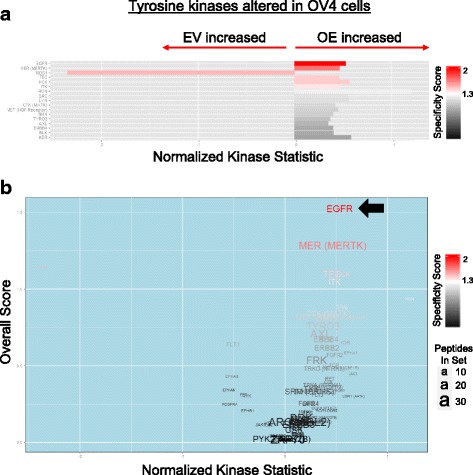
Tyrosine kinases, including EGFR, are more activated in cells with ST6Gal-I overexpression. Bar and volcano plots of kinases with altered PTK activity in OV4 OE vs. EV cells. **a.** Bar plot of tyrosine kinases identified with the BioNavigator UpKin PTK PamApp v8.0 (PamGene) scored as increased (rightward) or decreased (leftward) in OE relative to EV. Length of bar indicates extent of change (KSTAT; Kinase Statistic) and color of bar indicates specificity of each kinase to the predicted phosphosites used to measure its respective activity. **b.** In the volcano plot, kinases are similarly scored as increased or decreased (y axis) in OE relative to EV, colored by specificity. A combined KSTAT + specificity score (x axis) is denoted, with text size indicating the number of seed peptides used to identify that kinase

We next evaluated the activity of specific serine/threonine kinases modulated by ST6Gal-I (Fig 2). In general, most of the serine/threonine kinases probed by the kinomics assay exhibited increased activation in EV cells. The most prominent differentially activated kinases are shown in Fig. 2a. These include several members of the protein kinase C (PKC) and calcium-calmodulin-kinase-like (CAMKL) families. The specificity score measures the specificity of the kinase for its cognate peptides on the chip, with red indicating high specificity. The volcano plot in Fig. 2b relates the specificity score to the normalized kinase statistic score (i.e. relative kinase activity) to provide an overall score for kinase activation. Fig 3 depicts tyrosine kinase activity in EV and OV4 cells. Notably, nearly all of the tyrosine kinases screened were more active in OE cells (Fig. 3a). Many of these hyperactivated kinases are known to be associated with cell transformation, such as the nonreceptor tyrosine kinases, src and lyn, and receptor tyrosine kinases, MET, ERBB4 and EGFR. As shown in the volcano plot (Fig. 3b), one of the most differentially-activated tyrosine kinases in OE vs. EV cells was EGFR (arrow).

### ST6Gal-I overexpression in OV4 ovarian cancer cells increases basal and EGF-induced EGFR activation

**Fig. 4:**
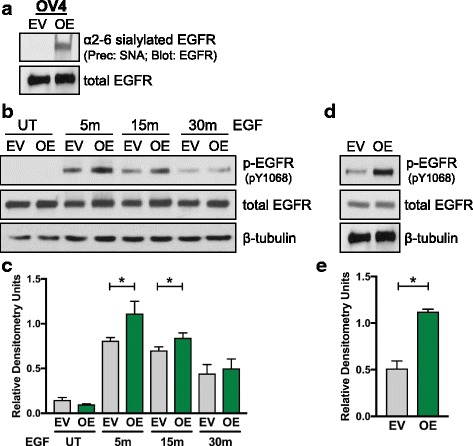
EGFR phosphorylation is enhanced in OV4 ovarian cancer cells with ST6Gal-I overexpression. **a.** To measure levels of α2-6 sialylated EGFR, cell lysates were incubated with SNA-agarose. SNA is a lectin that specifically recognizes α2-6 sialic acids. Sialylated proteins were precipitated and then immunoblotted for EGFR. **b.** Representative p-EGFR immunoblot of EV or OE cells treated with EGF for 5, 15 and 30 min. **c.** Densitometric analysis of three independent blots of p-EGFR in EGF-treated OV4 cells, with values normalized to the β-tubulin loading control. **d.** Representative p-EGFR immunoblot of EV or OE cells to evaluate basal EGFR phosphorylation. **e.** Densitometric analysis of three independent blots of basal p-EGFR in OV4 cells. *, *p* < 0.05

Given that the kinomics assay pointed to enhanced EGFR activation in cells with forced ST6Gal-I expression, we conducted additional analyses of this receptor. We first determined whether EGFR was a direct substrate for ST6Gal-I. To this end, cell lysates were incubated with agarose-conjugated SNA lectin to precipitate α2-6 sialylated proteins. These proteins were resolved by SDS-PAGE and immunoblotted for EGFR. As shown in Fig. 4a, α2-6 sialylated EGFR was clearly apparent in OE, but not EV, cells. The total amount of EGFR was equivalent in EV and OE cells, indicating that forced expression of ST6Gal-I did not alter EGFR expression. To assess the effects of EGFR sialylation on EGFR activation, cells were treated with EGF for up to 30 min, and then lysates were immunoblotted for p-EGFR (representative blot in Fig. 4b and corresponding densitometric analyses of blots from three independently-generated cell lysates in Fig 4c). At both 5 and 15 min following EGF stimulation, OE cells exhibited increased activation of EGFR relative to EV cells. In the blot depicted in Fig. 4b, p-EGFR levels in the untreated (UT) populations were below the limits of detection. We therefore optimized immunoblotting conditions to increase sensitivity (see Methods section), enabling visualization of basal p-EGFR. Fig. 4d (densitometry in Fig. 4e) shows that EGFR is more activated in the OE line even in the absence of exogenously-added EGF. These data are consistent with the kinomics assay, which was conducted on untreated cells.

### ST6Gal-I activity regulates basal and EGF-induced EGFR activation in SKOV3 ovarian cancer cells

**Fig. 5:**
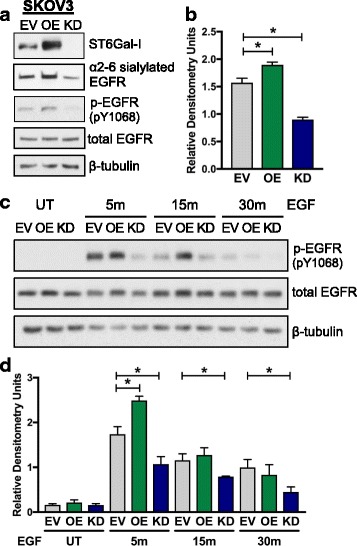
EGFR phosphorylation is enhanced in SKOV3 ovarian cancer cells with ST6Gal-I overexpression, but decreased in cells with ST6Gal-I knockdown. **a.** SKOV3 cells were stably transduced with lentivirus encoding human ST6Gal-I, or alternatively, shRNA for ST6Gal-I. ST6Gal-I overexpression (OE) and knockdown (KD) were confirmed by immunoblotting. EV, OE or KD cell lysates were precipitated by SNA-agarose and immunoblotted for EGFR to detect levels of α2-6 sialylated EGFR. Total cell lysates were immunoblotted for activated EGFR (p-EGFR, pY1068) or total EGFR. **b.** Densitometric analysis of three independent blots of basal p-EGFR in SKOV3 cells (normalized to β-tubulin). **c.** Representative p-EGFR immunoblot of EV, OE or KD cells treated with EGF for 5, 15 and 30 min. **d.** Densitometric analysis of three independent blots of p-EGFR in EGF-treated SKOV3 cells. *, *p* < 0.05

To determine whether sialylation-dependent regulation of EGFR was conserved across cancer cell lines, EGFR activity was examined in the SKOV3 ovarian cancer line. ST6Gal-I was either overexpressed (OE) in SKOV3 cells, or stably knocked-down (KD) using a shRNA-bearing lentiviral vector (Fig. 5a). SNA precipitation assays showed that, in comparison with EV control cells, EGFR had increased α2-6 sialylation in OE cells, and decreased α2-6 sialylation in KD cells. As with the OV4 line, manipulating ST6Gal-I expression in SKOV3 cells had no effect on total levels of EGFR. In correspondence with differential sialylation, basal p-EGFR was higher in SKOV3 OE vs. EV cells, whereas p-EGFR levels were greatly reduced in KD cells (densitometry for p-EGFR in Fig. 5b). Along with changes in basal activation, sialylation of EGFR regulated the response to EGF (representative blots in Fig. 5c and densitometry in Fig. 5d). EGF-induced EGFR activation was enhanced in OE cells, but diminished in KD cells.

### ST6Gal-I knockdown impairs basal and EGF-induced EGFR activation in BxPC3 pancreatic cancer cells

**Fig. 6:**
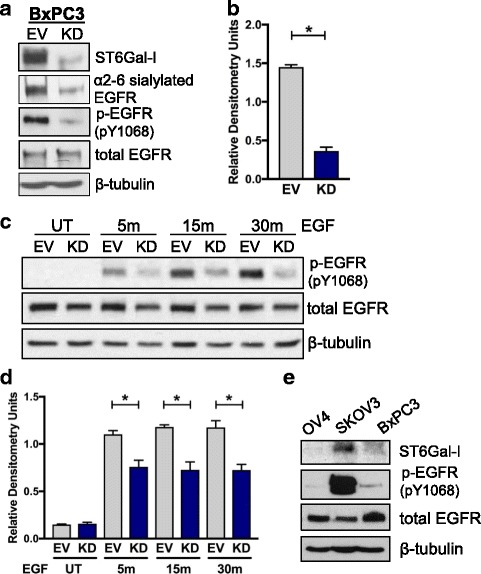
Knockdown of ST6Gal-I diminishes EGFR phosphorylation in BxPC3 pancreatic cancer cells. **a.** Using lentivirus, BxPC3 cells were stably transduced with shRNA for ST6Gal-I, and ST6Gal-I knockdown (KD) was confirmed by immunoblotting. EV or KD cell lysates were precipitated by SNA agarose and immunoblotted for EGFR to detect α2-6 sialylated EGFR. Total cell lysates were immunoblotted for activated EGFR (p-EGFR, pY1068) or total EGFR. **b.** Densitometric analysis of three independent blots of basal p-EGFR in BxPC3 cells (normalized to β-tubulin). **c.** Representative p-EGFR immunoblot of EV or KD cells treated with EGF for 5, 15 and 30 min. **d.** Densitometric analysis of three independent blots of p-EGFR in EGF-treated BxPC3 cells. **e.** Total cell lysates from EV cells of the OV4, SKOV3 and BxPC3 lines were immunoblotted for ST6Gal-I, p-EGFR (Y1068) or total EGFR. *, *p* < 0.05

Recent studies suggest that ST6Gal-I has a similar function in multiple cancer types including ovarian, colon, and pancreatic adenocarcinoma. We therefore evaluated EGFR activation in a pancreatic cancer line, BxPC3. ST6Gal-I expression was knocked down (KD) in this line, which led to a loss in α2-6 sialylation of EGFR, as measured by SNA precipitation assay (Fig. 6a). Consistent with reduced EGFR sialylation, ST6Gal-I KD cells had dramatically decreased levels of basal activation of EGFR (densitometry for p-EGFR in Fig. 6b). As with basal activation, EGF-stimulated EGFR activation was suppressed by ST6Gal-I KD (representative blots in Fig. 6c and densitometry in Fig. 6d). Considering that elevated ST6Gal-I levels resulted in increased EGFR activation in all three cell lines, we next tested whether endogenous expression of ST6Gal-I correlates with p-EGFR in a side-by-side comparison of the lines. Fig. 6e shows that SKOV3 EV cells, which have the highest levels of ST6Gal-I of the three lines, exhibit the most pronounced basal activation of EGFR, whereas the lowest ST6Gal-I expressing cells, OV4 EV, display the least amount of basal p-EGFR. This finding suggests that, irrespective of the diverse genetic backgrounds of these three cell lines, there is correspondence between the level of ST6Gal-I activity and EGFR activation.

### ST6Gal-I-mediated sialylation of EGFR regulates the viability of cells treated with gefitinib

**Fig. 7:**
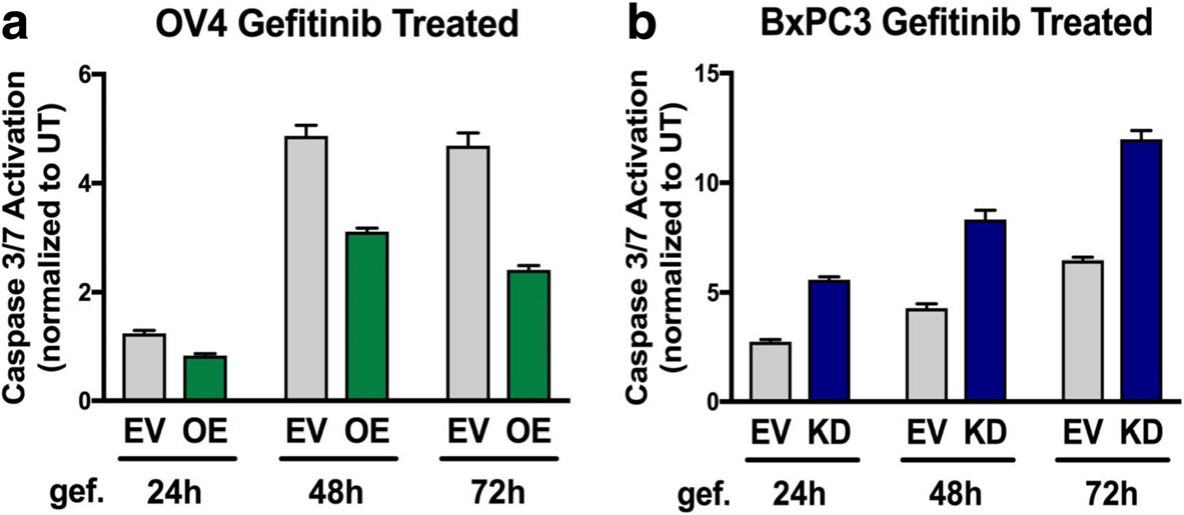
ST6Gal-I activity protects cancer cells against gefitinib-mediated apoptosis. **a.** OV4 EV or OE cells and **b.** BxPC3 EV or KD cells were treated with gefitinib for 24, 48 and 72 h and analyzed for apoptosis via a luminescence assay that detects caspase 3 and 7 activity. Caspase activity in geftinib-treated cells was normalized to caspase activity in untreated cells. Data shown are from a representative experiment, with at least 2 independent experiments performed for each cell line. Graph depicts means ± S.D

The combined results in Figs 1, 2, 3, 4, 5 and 6 established that ST6Gal-I mediated sialylation of EGFR enhances its activation, as indicated by greater tyrosine kinase activity. To confirm the importance of ST6Gal-I in regulating EGFR-dependent cell survival, we examined cell response to the EGFR inhibitor, gefitinib. In a prior study, it was shown that ST6Gal-I KD sensitizes cells to gefitinib-induced cell death [27]. Accordingly, OV4 EV and OE cells were treated with gefitinib, and then monitored for apoptosis using a caspase 3/7 activity assay. As shown in Fig. 7a, OV4 OE cells were resistant to gefitinib-mediated apoptosis. Contrarily, ST6Gal-I KD in BxPC3 cells enhanced gefitinib-induced cell death (Fig. 7b).

## Discussion

Accumulating evidence suggests that ST6Gal-I is a potent survival factor, providing cancer cells with protection against a variety of microenvironmental assaults. Explicitly, α2-6 sialylation of select receptors inhibits galectin-mediated apoptosis [28–30], and ST6Gal-I also prevents cell death driven by TNFR1 [14, 31] and Fas [13]. In addition, ST6Gal-I protects tumor cells against cytotoxicity mediated by radiation [32] and chemotherapy drugs including cisplatin, gemcitabine and docetaxel [5, 17–19]. Furthermore, ST6Gal-I enables tumor cell survival under conditions of serum growth factor deprivation by enhancing Akt activation, and preventing tumor cell exit from the cell cycle [26].

In the current study, we describe a new survival function for ST6Gal-I in modulating the activity of EGFR. Utilizing an unbiased kinomics assay, we show that overall basal tyrosine kinase activity, a phenomenon well-known to contribute to malignant transformation [33–36], is profoundly elevated in cells with forced overexpression of ST6Gal-I. In particular, EGFR is one of the most highly activated of these tyrosine kinases. To substantiate this result, we manipulated ST6Gal-I expression in 3 different cancer cell lines, using both overexpression and knockdown approaches. In these models, EGFR was shown to be a direct substrate for ST6Gal-I-mediated sialylation, and in every case, α2-6-sialylation of EGFR increased both the basal and EGF-induced activation of EGFR. Thus, EGFR α2-6-sialylation is consistently correlated with increased tyrosine kinase activity despite the diverse genetic lesions present in the 3 distinct cancer lines. As well, ST6Gal-I-mediated EGFR sialylation protected tumor cells against gefitinib-induced cell death.

The role of sialylation in regulating EGFR has been previously investigated. Park et al. manipulated ST6Gal-I expression in colon carcinoma cells, and in concordance with our work, reported that EGFR sialylation prevented geftinib-induced cytotoxicity [27]. However, in contrast to our studies, α2-6 sialylation appeared to inhibit EGF-induced EGFR tyrosine phosphorylation [27]. The reason for this discrepancy is currently unclear. Other investigators have manipulated global sialylation to interrogate the functional effects on EGFR. Wong’s group incubated cells with a sialidase enzyme that cleaves all sialic acid linkages (α2-3, α2-6 and α2-8) [37]. In this study, the removal of surface sialylation facilitated the formation of EGF-induced EGFR dimers. This group further generated a recombinant, soluble EGFR protein and reported that sialidase-treated EGFR protein exhibited greater dimerization [38]. Yarema’s group addressed the question of EGFR sialylation by incubating cells with a metabolic precursor of sialic acid that augments the intracellular pool of sialic acid, leading to enriched receptor sialylation [39]. The ensuing increase in EGFR sialylation was found to hinder EGFR’s association with the extracellular galectin lattice, potentiating EGFR internalization. No changes in EGFR dimerization were observed in this latter study. Taken together, these studies highlight the importance of sialic acid in EGFR signaling, however there are some caveats associated with manipulating the global sialylation of tumor cells. First, complete removal of cell surface sialylation via sialidase treatment has profound effects on cell signaling, altering the activity of a myriad of receptors. Secondly, there is substantial evidence that the α2-3 and α2-6 sialic acid linkages are not always functionally equivalent. This is best exemplified by the activity of lectins such as galectins and siglecs that clearly discriminate between α2-3 and α2-6 sialylation. Finally, there is an extensive literature suggesting that α2-6 sialylation is particularly enriched in cancer cells [1–3] (and also stem cells [40, 41]). Our group has sought to model the tumor cell phenotype by examining the effect of selective ST6Gal-I upregulation, without grossly altering α2-3 sialylation. Through this approach, we find that high ST6Gal-I activity enhances EGFR activation, consistent with the vast literature suggesting that ST6Gal-I acts as a tumor-driver gene.

Further studies will be needed to better understand the effect of α2-6 sialylation on EGFR. EGFR has a complex mechanism of regulation involving receptor oligomerization, lipid raft localization, shedding, and dynamic partitioning between cellular compartments including the plasma membrane, endosome and nucleus [42–45]. Many of these processes are known to be influenced by EGFR glycan composition [39, 46–48]. Furthermore, glycosylation modulates the overall conformation of EGFR in the absence of ligand binding. For example, the *N*-glycans on two key Asn residues, Asn-420 and Asn 579, appear to maintain EGFR in a low affinity state. Ablation of Asn-420 glycosylation (via mutagenesis) leads to constitutive EGFR tyrosine phosphorylation and spontaneous oligomer formation [49]. Elimination of the Asn-579 site weakens auto-inhibitory tether interactions, and increases the number of preformed EGFR dimers in the absence of ligand [50]. It is tempting to speculate that the addition of the bulky, negatively-charged sialic acid at Asn-420 and/or Asn-570 could disrupt these critical auto-inhibitory interactions, potentiating basal EGFR activation.

Regardless of the mechanism by which α2-6 sialylation modulates EGFR structure (or potentially, localization), results herein are consistent with other studies indicating that ST6Gal-I protects against gefitinib-induced cell death [27]. Gefitinib is widely-used in cancer therapy [51], and hence, the levels of ST6Gal-I expression in patient samples could be an important indicator of patient response to treatment. Intensive investigation is currently focused on how EGFR modifications affect the efficacy of EGFR inhibitors, however the role of EGFR glycosylation in drug response has received limited attention.

## Conclusions

The collective data in this report indicate that upregulation of ST6Gal-I, a common feature of cancer cells, promotes heightened activation of EGFR as well as resistance to gefitinib-induced cell death. Considering the immense contribution of EGFR to cancer [43, 52, 53], the establishment of α2-6 sialylation as a novel EGFR regulatory mechanism could advance a fundamental understanding of the relationship between growth factor signaling and cancer cell behavior.
